# Infection Control and SARS Transmission among Healthcare Workers, Taiwan

**DOI:** 10.3201/eid1005.030777

**Published:** 2004-05

**Authors:** Yee-Chun Chen, Pei-Jer Chen, Shan-Chwen Chang, Chiang-Lian Kao, Shiou-Hwa Wang, Li-Hua Wang, Pan-Chyr Yang, the SARS Research Group of National Taiwan University College of Medicine and National Taiwan University Hospital

**Affiliations:** * National Taiwan University Hospital, National Taiwan University College of Medicine, Taipei, Taiwan

**Keywords:** Infection-control measures, serologic assays, SARS-CoV

## Abstract

This study found infrequent transmission of severe acute respiratory syndrome (SARS) coronavirus to healthcare workers involved in the care of the first five case-patients in Taiwan, despite a substantial number of unprotected exposures. Nonetheless, given that SARS has been highly transmissible on some occasions, we still recommend strict precautions.

Healthcare workers may be unwittingly exposed to the severe acute respiratory syndrome–associated coronavirus (SARS-CoV) from patients with pneumonia at the onset of an epidemic (*1*,*2*). They are also at increased risk of acquiring SARS from known case-patients with a high viral load who require intensive respiratory care (*1*–*3*). The first case-patient in Taiwan was admitted to National Taiwan University Hospital on March 8, 2003, before the World Health Organization (WHO) issued the first global alert (*4*,*5*). The patient was intubated in the emergency room and was admitted to the intensive care unit. The second case-patient, his wife, was admitted to the emergency room with pneumonia on March 14. The occurrence of two cases of pneumonia in the same household within 6 days, together with the patients’ recent travel to Guangdong, China, through Hong Kong, led us to suspect a diagnosis of atypical pneumonia, which later came to be known as SARS.

Before the second case was detected, healthcare workers routinely used standard precautions. Specific infection-control measures, including droplet and contact precautions against SARS, were implemented after the second patient was admitted. The efficacy of these infection-control measures in protecting healthcare workers was determined by: 1) the occurrence of SARS symptoms as defined by WHO criteria (*6*) and 2) a rise in antibodies to SARS-CoV before and after specific infection-control measures were implemented.

## The Study

From March 8 to March 28, the hospital admitted five patients in whom SARS-CoV infection was subsequently laboratory-confirmed. The patients were isolated in negative-pressure rooms. Patients 2 and 3 were family members of patient 1. Patients 4 and 5 were believed to have contracted SARS on a March 15 flight from Hong Kong to Beijing. Four of the patients progressed rapidly to respiratory failure and were intubated.

Healthcare workers caring for these patients were exposed during two periods. During March 8–14, before specific infection-control precautions were implemented, 73 healthcare workers were exposed to patients 1 and 2. During March 15–28, after specific precautions were implemented, an additional 150 healthcare workers were exposed to all five patients.

All healthcare workers who had contact with SARS patients used personal protective equipment, including gown, gloves, N-95 respirators, disposable cap, and shoe covers. Healthcare workers exposed to SARS patients or their environments were monitored for signs or symptoms of SARS for 14 days after the last exposure. Healthcare workers who had high-risk exposures to SARS were excluded from new duty assignments. We considered performing any of the following to be a high-risk exposure: endotracheal intubation >30 min, cardiopulmonary resuscitation >30 min, pleurocentesis >30 min, or bedside care (such as chest care [including percussion and postual drainage] or feeding) >30 min. Any healthcare worker in whom fever developed (temperature >38°C) was isolated in a specially designated ward.

A total of 223 healthcare workers exposed to SARS patients were interviewed by one of two researchers with a structured questionnaire designed by the Centers for Disease Control and Prevention (CDC), USA, and the Center for Disease Control, Taiwan. The following data were recorded on uniform case-report sheets: extent of personal protective equipment use during exposure, type of exposure (stay in the same room, direct patient contact, or exposure to respiratory droplets and secretions), disease phase of patients to whom they were exposed (during incubation period, early fever, fever and cough, or intubation period), occurrence of fever (>38°C), and respiratory or gastrointestinal symptoms after exposure. Proportional data were tested by using χ^2^ or Fisher exact test (EpiInfo 6, CDC, Atlanta, GA). A p value <0.05 was considered significant. The Ethics Committee of the hospital approved these studies.

Serum samples were collected twice from 206 healthcare workers during a 1-month period after the initial exposure to patients with SARS, with a minimum interval between collections of 2 weeks. Serologic response to SARS-CoV was determined by using an indirect immunofluorescence assay (IFA) as described previously (*5*) and the immunochromatographic test (ICT, Tyson BioResearch, Inc, Taiwan). ICT consists of a double-antigen (recombinant viral nucleocapsid antigen) sandwich. The test gives results within 15 min. Data obtained from 13 patients with severe SARS, as defined by using CDC criteria (*7*), showed that the sensitivity of the ICT test was >90% within 2 weeks of fever onset and 100% after 6 weeks. Data obtained from 51 cases of severe SARS demonstrated that the sensitivity of either IFA or ICT was 98% after 6 weeks (*8*). Furthermore, the specificity of each assay determined by 812 serum samples was 100%.

The Table compares the extent of personal protective equipment use before and after implementing specific infection-control measures. Healthcare workers during the “after” period were substantially more likely than the “before” period to have used full personal protective equipment (Table).

**Table:** Personal protection before and after recognizing severe acute respiratory syndrome (SARS) and implementing specific infection-control measures at the National Taiwan University Hospital

	Exposure type								
	In the same room^a^			Direct contact			Exposure to respiratory droplets and secretions		
Protective measures	Before (n = 73)	After (n = 155)	p value	Before (n = 46)	After (n = 132)	p value	Before (n = 37)	After (n = 92)	p value
Masks			<0.001			<0.001			<0.001
None	36	0		20	0		17	0	
Surgical mask, N-95 or P-100 respirator	37	155		26	132		20	92	
Gloves			<0.001			<0.001			<0.001
None	57	7		28	4		17	2	
One- or two-layer	16	148		18	128		20	90	
Eye protection			<0.001			<0.001			<0.001
No	73	117		46	99		37	66	
Glasses, goggles, or face shields	0	38		0	33		0	26	
Gowns			<0.001			<0.001			<0.001
None	66	6		38	6		30	3	
One- or two-layer	7	149		8	126		7	89	

^a^Five healthcare workers stayed in the same room with SARS patients before and after implementation of specific infection-control measures. Among 223 healthcare workers, 178 had direct contract to SARS patients or their environment, and 129 had exposure to respiratory droplets and secretion.

First serum samples were collected 12.4 ± 5.4 days (mean ± standard deviation) after initial exposure to SARS patients. Second serum samples were collected 37.2 ± 7.9 days after exposure. Ninety percent were collected >30 days after exposure. None of the 73 healthcare workers exposed during the before period produced a positive result on serologic tests for SARS. This group included a physician who intubated patient 1 and wore two layers of surgical masks and used inline suction after intubation. SARS developed in 1 of 150 healthcare workers exposed during the after period. This healthcare worker was a chest physician. On March 17, he performed a 30-min chest sonogram on patient 2 in a negative-pressure isolation room and wore an N-95 respirator, double gloves, gown, disposable cap, and shoe covers. On the same day, he helped intubate patient 2 while positioned approximately 3 feet from the patient’s head. During this period, patient 2 was irritable and had a vigorous cough. The physician recalled that he had not tried the mask on or confirmed that it was air-tight before entering the isolation room. Fever developed 4 days later in this physician, designated as patient 6, and pneumonia developed 5 days after that. Both virus culture and reverse transcriptase–polymerase chain reaction (RT-PCR) demonstrated SARS-CoV in the sputum. Immunoglobulin (Ig) G against SARS-CoV determined by IFA was >1:1,000 (*5*). After this experience, the infection-control team reemphasized the importance of fit-testing facemasks and recommended wearing a face shield when in close contact with SARS patients. SARS did not develop in another physician who intubated patient 2 and four nurses who assisted the procedure in the same room.

## Conclusions

In this study, a physician who intubated a patient with SARS while following standard precautions did not become ill, but SARS developed in another physician whose N-95 respirator was not properly fit-tested. A serologic response to SARS-CoV could not be demonstrated in 205 healthcare workers who spent time in the same room as or had direct contact with SARS patients.

The major question that arises from this study is why 36 (50%) healthcare workers who stayed in the same room with SARS patients before the outbreak was recognized and who did not wear masks were not infected. Several possible explanations exist. Patient 2 wore a face mask when she visited the emergency room. The physician who intubated patient 1 was alert, wore two layers of surgical masks, and followed standard precautions. Inline suction was routinely performed at the hospital for intubated patients to prevent aerosol formation; therefore, unprotected healthcare workers might not have been exposed to a sufficient amount of SARS-CoV to produce a systemic infection. An alternate explanation could be that existing serologic assays are not sufficiently sensitive to identify subclinical infections. This explanation is unlikely, however, because the tests we used have been shown to be highly sensitive and specific in patients with SARS (*5*,*8*), and 90% of convalescent-phase serum samples were collected >30 days after exposure. Yet another explanation could be that SARS-CoV is attenuated by serial passage in humans. This explanation is also unlikely since SARS developed in the five index patients admitted to the hospital in the early phase of the epidemic and in one physician with a poorly fitting mask. Further, phylogenic tree analysis (*9*) indicates that patients 2, 3, and 6 were infected by strains related to the large outbreak in Amoy Gardens in Hong Kong (*2*), and patients 4 and 5 were infected by strains related to a large hospital outbreak in Taipei (*10*). A final explanation could be, simply, that the disease does not develop in all people exposed to the virus.

Transmission of SARS was limited initially at our hospital (attack rate 0.4%) when healthcare workers followed standard precautions or specific infection-control measures, including droplet and contact precautions. However, in later stages of the epidemic, SARS was more likely to develop in healthcare workers, despite similar or higher levels of personal protective equipment use. Although one possible explanation for this could have been exposure to unrecognized SARS patients, contamination of the environment leading to indirect contact transmission may have also played a role (*11*).

In conclusion, while SARS-CoV can spread rapidly in a nonimmune human population (*1*–*3*), this study demonstrated infrequent transmission of SARS to healthcare workers caring for the first five SARS patients in Taiwan, despite a number of unprotected exposures. Nonetheless, given that SARS has, on other occasions, shown itself to be highly transmissible (*1*–*3*,*10*), we still recommend strict precautions (*1*–*3*,*11*–*14*).
